# Cocaine

**Published:** 1886-05

**Authors:** 


### COCAINE.

When hydrochlorate of cocaine was first introduced in medicine the visionary enthusiasts at once declared that man was now master of all pain. They exaggerated its powers and, without knowledge of its full nature and properties, ignorant of the best way to manufacture or use it, they made applications to all tissues alike. Their solutions would not keep a week, and all sorts of adulterations were used. What wonder then that a reaction came and that cocaine was cast out?

We have lately been experimenting further with the drug, and are pleased with the results. Some of the beautiful crystals of Parke, Davis & Co., the manufacturing chemists, of Detroit, were procured, and fresh solutions made at the time of using. Injecting a four per cent. solution about the roots of an extremely painful and firmly set molar stump, after a prolonged struggle it was removed, quite without pain. We have operated on pyorrhœal cases, have removed living pulps, opened abscesses and performed other operations painlessly. With a ten per cent. solution we have obtunded very sensitive dentine, in the majority of cases.

So satisfactory were the results, that one of Parke, Davis & Co.'s cocaine cases, containing every requisite for the proper preparation and application of cocaine, was procured, and thus we are armed for every case which may present itself. Further knowledge of the character of the drug has convinced us that it is an essential in the case of every operative dentist who regards the comfort of his patients. Of all the manufactures there is none more reliable than that of Parke, Davis & Co.

